# Visualizing cortical laminar architecture in the living human brain using next-generation ultra-high-gradient diffusion MRI

**DOI:** 10.21203/rs.3.rs-6724971/v1

**Published:** 2025-06-10

**Authors:** Susie Huang, Hansol Lee, Yixin Ma, Kwok-Shing Chan, Eva Krijnen, Laleh Eskandarian, Aneri Bhatt, Julianna Gerold, Mirsad Mahmutovic, Oula Puonti, Xiangrui Zeng, Lucas Jacob Deden Binder, Bruce Fischl, Boris Keil, Gabriel Ramos-Llordén, Eric Klawiter, Hong-Hsi Lee

**Affiliations:** Athinoula A. Martinos Center for Biomedical Imaging / Massachusetts General Hospital; Harvard Medical School; Mittelhessen University of Applied Science; Massachusetts General Hospital

**Keywords:** Cortical microstructure, Laminar organization, Diffusion MRI, High-performance gradient, Connectome 2.0, SANDI Model, Cytoarchitecture, Myeloarchitecture

## Abstract

Characterizing cortical laminar microstructure is essential for understanding human brain function. Leveraging the next-generation Connectome MRI scanner (maximum gradient strength = 500mT/m, slew rate = 600T/m/s), we characterized *in vivo* cortical laminar cytoarchitecture and myeloarchitecture through cortical depth-dependent analyses of soma and neurite density imaging (SANDI) metrics derived from diffusion MRI, enhanced by a super-resolution technique. SANDI revealed distinct laminar profiles: intra-soma signal fraction *f*_*is*_ peaked at ~ 55% cortical depth, while intra-neurite signal fraction *f*_*in*_ increased toward deeper layers, consistent with histological patterns. The visual cortex exhibited higher intra-soma signal fraction *f*_*is*_ than the motor cortex, particularly in deeper layers. Moreover, intra-soma signal fraction *f*_*is*_ correlated positively with cortical curvature in superficial layers and negatively in deeper layers, indicating layer-specific relationships between microstructure and cortical geometry. These findings demonstrate the feasibility of noninvasively mapping cortical laminar architecture, offering a potential surrogate for histology and enabling future studies of normative and pathological brain organization using commercially available high-performance gradient MRI systems.

## Introduction

The cerebral cortex exhibits diverse and intricate cyto- and myeloarchitectonic organization underpinning motor, sensory, cognitive, and other functions unique to humans^1–6^. The size and density of neuronal and glial cell bodies as well as myelin content exhibit distinct profiles across cortical depths, a characteristic known as laminar specificity^7,8^. Cortical laminar patterns can be broadly categorized into three compartments: supragranular layers (I-III), granular layer (IV), and infragranular layers (V-VI), comprising a total of six distinct layers (I-VI)^9–11^. Each layer is characterized by unique molecular, cellular, and connectivity profiles. The brain’s connections within and between cortical columns are organized in specific patterns that support hierarchical information processing. These structural and connectivity patterns form the basis for the functional specialization of different cortical areas within neural circuits^1,2,5,11^. Characterizing the cyto- and myeloarchitectonic features in the living human brain is fundamental to elucidating their contributions to cognition, sensory processing, and motor coordination, as well as their involvement in a variety of neuropsychiatric disorders.

The detailed examination of brain tissue microstructure has traditionally relied on postmortem tissue analysis using microscopic imaging techniques, including histological staining, electron microscopy, and immunofluorescence^12–14^, which enable the direct characterization of cellular components and their organization. *Ex vivo* magnetic resonance imaging (MRI) is a powerful tool for imaging larger areas of postmortem brain specimens than traditional histological techniques, providing imaging of whole brain specimens down to 100 pm in resolution without the need for tissue sectioning^15–18^. *Ex vivo* MRI reveals detailed neuroanatomical features, such as cortical lamination^19^, hippocampal subfield architecture^20^, and white matter organization^21^, as well as pathological protein deposition like the distribution of amyloid plaques^22^ and vascular abnormalities^23^ associated with neurodegenerative diseases. While these approaches have greatly contributed to our understanding of brain anatomy and pathology, their inability to capture physiological processes or functional connectivity, coupled with artifacts introduced by tissue fixation and processing^24^, limits their utility in studying the dynamic aspects of the living brain. The development and advancement of *in vivo* neuroimaging techniques^25–29^, driven by innovations in MRI technology, are essential for bridging the gap between postmortem histology and translational research, enabling unprecedented exploration of the living human brain’s microstructural features, real-time physiological processes, and functional connectivity. Anatomical MRI is effective in identifying macroscopic features like large-scale brain organization and structural abnormalities, such as atrophy or lesions^30–32^. Diffusion MRI (dMRI) complements these structural insights by probing tissue features at the cellular level, enabling the detection of subtle changes in tissue microarchitecture, including alterations in axonal integrity, synaptic density, and cellular composition^33–35^.

The capability of dMRI to investigate brain tissue microstructural organization in the living human brain stems from its measurement of the random Brownian motion of water molecules within tissues^36–38^. The accuracy of imaging tissue micro-geometries using dMRI relies on the maximum strength of magnetic field gradients used during the scanning process^39–41^. One of the key recent innovations in enabling the characterization of brain tissue microstructure in the living human brain has been the development of high-performance gradient MRI systems with magnetic field gradient strengths on the order of hundreds of mT/m for *in vivo* human imaging^26–29^. Such high-performance gradient MRI scanners exceed the capabilities of conventional clinical MRI scanners equipped with lower gradient strengths ~ 40–80 mT/m. The first-generation 3 Tesla Human Connectome MRI scanner was equipped with a whole-body gradient system capable of reaching a maximum gradient strength (*G*_max_) of 300 mT/m^42^. This pioneering system made substantial progress in the mapping of white matter connections^43^, axonal diameters^44–47^, and intra-soma and intra-neurite density across the human lifespan^48^. Despite these advances, we and others have shown that pushing the maximum gradient strength beyond 300 mT/m on the original Connectome MRI scanner (Connectome 1.0) enables unprecedented *in vivo* investigations of brain microstructure^39–41^.

The recently developed next-generation Connectome MRI scanner (Connectome 2.0) represents a significant leap forward in MRI capabilities for tissue microscopic imaging^26,29^. Equipped with a *G*_max_ of 500 mT/m and a maximum slew rate (*SR*_max_) of 600 T/m/s, Connectome 2.0 provides a unique opportunity to probe cortical laminar architecture in the living human brain with higher microstructural resolution than previously attainable. The implementation of stronger gradients and higher slew rates brings multiple advantages for microstructural imaging using dMRI^47,49–51^, such as shorter diffusion times and gradient durations, and subsequently shorter echo times (TE) during the image acquisition, resulting in higher signal-to-noise ratio and higher spatial resolution as well as improved microstructural specificity and sensitivity to the complex tissue properties of the living human brain at finer scales^26,29,42^. Combined with advanced biophysical models of dMRI, such as the Soma and Neurite Density Imaging (SANDI) model^51^, diffusion measurements using strong gradients enable us to disentangle signal contributions from multiple cellular components, including the cell body (soma), neurites, and extracellular space. As such, Connectome 2.0 provides a powerful tool for noninvasive mapping of gray matter microstructure in the human brain. The ability to differentiate between soma and neurite components allows a more detailed examination of cortical cytoarchitecture and myeloarchitecture, providing more specific, histological-level information than current *in vivo* imaging methods^49,52^. In our recent study on normal brain aging, we applied the SANDI model to data from the Connectome 1.0 scanner and demonstrated age-related reductions in cortical cell body density that closely track with cortical volume loss in brain regions known to be affected in normal aging^53^. Additionally, in individuals with multiple sclerosis, we observed a significant loss of cortical cell body density in lesions that correlates with subregional thalamic volume loss^48,54,55^.

The goal of this study was to advance our understanding of cortical microstructure in the living human brain by leveraging the enhanced diffusion encoding capabilities of the Connectome 2.0 scanner. We first analyzed SANDI metrics derived from dMRI acquired in healthy young adults, focusing on the supragranular and infragranular layers, then conducted a thorough cortical-depth-dependent analysis to map the cytoarchitectonic and myeloarchitectonic organization across multiple cortical depths. We sought to reveal laminar and regionally specific variations in microstructure, facilitating the characterization of cortical areas based on their unique cytoarchitectonic and myeloarchitectonic properties, and compared dMRI results to established histological atlases^8,56,57^. In addition, to assess the impact of gradient hardware advancements on the derived cortical microstructural metrics, we performed a comparative study of SANDI metrics obtained from protocols carried out on the Connectome 2.0 and Connectome 1.0 scanners in age- and sex-matched healthy young adults. The enhanced diffusion encoding capabilities of the Connectome 2.0 scanner resulted in shorter diffusion times and TE, leading to higher signal sensitivity to restricted diffusion within cellular compartments, particularly within neurites, enhancing the specificity of *in vivo* brain microstructure imaging compared to the results obtained with Connectome 1.0.

## Results

**Figure 1:**
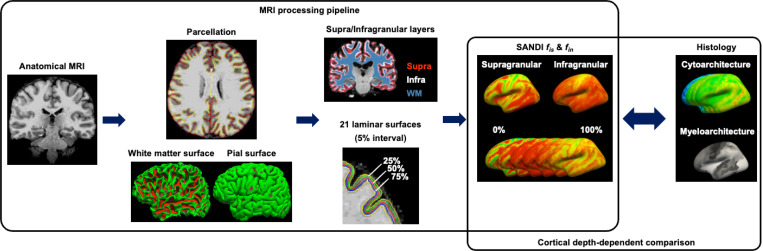
Framework for cortical depth-dependent microstructure analysis of SANDI metrics, with comparisons to histological atlases.

We applied a framework using a multi-step analysis pipeline (Figure 1) that includes anatomical parcellation, surface reconstruction, and laminar sampling of SANDI metrics (intra-soma signal fraction *f*_*is*_ and intra-neurite signal fraction *f*_*in*_*)* to assess cortical microstructural features in the living human brain and compare them with cytoarchitectonic and myeloarchitectonic histological atlases.

### Cohort characteristics by scanner

A total of 42 healthy adults under 40 years of age participated in this study at Massachusetts General Hospital. Participants were divided into two groups: 21 individuals (14 females, 7 males; mean age: 29.0±4.5 years; age range: 19–37) underwent MRI scans using the newly installed 3T Connectome 2.0 MRI scanner (*G*_max_ of 500 mT/m and maximum slew rate of 600 T/m/s). Age- and sex-matched 21 participants (14 females, 7 males; mean age: 28.7±6.2 years; age range: 19–40) were scanned on the 3T Connectome 1.0 scanner (*G*_max_ of 300 mT/m and maximum slew rate of 200 T/m/s).

### Self-similarity-based super-resolution imaging processing technique

The high-resolution (1 mm isotropic) dMRI-derived SANDI metrics generated using the super-resolution technique^58–60^ improved the visualization of detailed microstructural features and effectively reduced partial volume effects compared to the lower-resolution data (Supplementary Figure 1; see Supplementary Note 1 for details on the super-resolution imaging processing technique).

### SANDI metrics comparison between Connectome 2.0 and Connectome 1.0

The SANDI metrics across the full cortical depth averaged across subjects for each scanner are displayed on the FreeSurfer “fsaverage” template space in Supplementary Figure 2. The intra-neurite signal fraction *f*_*in*_ was significantly higher on the Connectome 2.0 scanner (mean = 0.21 ± 0.01) compared to the Connectome 1.0 scanner (mean = 0.16 ± 0.01; FDR-*P*<0.001). In contrast, intra-soma signal fraction *f*_*is*_ showed no significant difference between the two scanners (mean = 0.43 ± 0.02 *vs.* 0.43 ± 0.01; FDR-*P*=0.35).

### SANDI metrics across cortical depths

**Figure 2:**
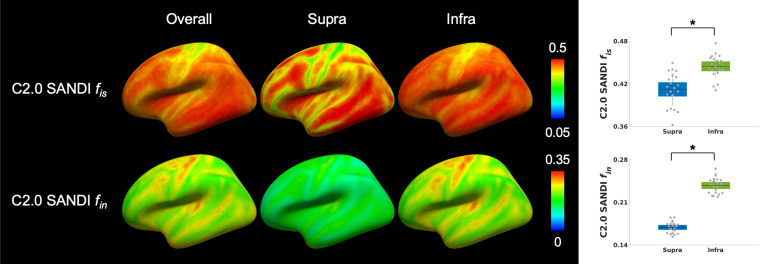
SANDI-derived microstructural metrics on the Connectome 2.0 (C2.0) scanner in supragranular (Supra) and infragranular (Infra) layers. **Left:** Cortical maps of the intra-soma signal fraction *f*_*is*_ and intra-neurite signal fraction *f*_*in*_ derived from SANDI, averaged across 21 individuals. **Right:** Boxplots summarizing intra-soma signal fraction *f*_*is*_ and intra-neurite signal fraction *f*_*in*_ from data of 21 individuals, with statistically significant differences (*: FDR-*P*<0.05) between supragranular and infragranular layers. The box represents the 1.96 standard error of the mean (95% confidence interval), and the line represents the 1 standard deviation.

Cortical distribution and layer-specific differences in microstructural metrics derived from the SANDI model on the Connectome 2.0 scanner are illustrated in Figure 2. Cortical surface maps showed the intra-soma signal fraction *f*_*is*_ and intra-neurite signal fraction *f*_*in*_ across the entire cortex (Overall), as well as separately within supragranular (Supra) and infragranular (Infra) layers. These laminar profiles revealed significantly higher intra-soma signal fraction *f*_*is*_ and intra-neurite signal fraction *f*_*in*_ in the infragranular layers (mean *f*_*is*_ = 0.44±0.02, mean *f*_*in*_ = 0.24±0.01) compared to the supragranular layers (mean *f*_*is*_ = 0.41±0.02, mean *f*_*in*_ = 0.17±0.01), with both comparisons showing FDR-*P*<0.001.

The utility of laminar-specific metrics to distinguish adjacent cortical regions is illustrated in Supplementary Figure 3. While the overall intra-soma signal fraction *f*_*is*_ did not differ significantly between motor cortex subregions BA4a and BA4p (BA4a: 0.39±0.03., BA4p: 0.39±0.03; FDR-*P*=n.s.), layer-specific comparisons revealed significant differences between supra- and infragranular estimates of intra-soma signal fraction *f*_*is*_. In the supragranular layer, intra-soma signal fraction *f*_*is*_ was significantly lower in BA4a compared to BA4p (mean = 0.36±0.03 *vs.* 0.39±0.04; FDR-*P*<0.001). Conversely, infragranular intra-soma signal fraction *f*_*is*_ was higher in BA4a than in BA4p (mean = 0.41±0.02 *vs.* 0.40±0.03; FDR-*P*<0.001).

**Figure 3:**
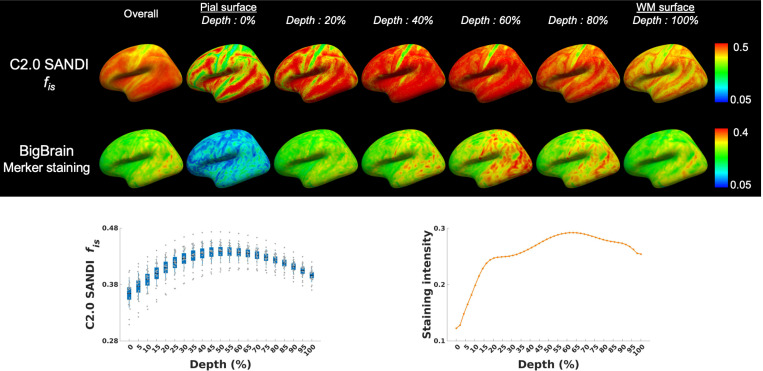
SANDI-derived intra-soma signal fraction *f*_*is*_ on the Connectome 2.0 scanner, alongside Merker staining data from the Bigbrain atlas for cytoarchitecture across the cortical depths. The data of BigBrain Merker staining intensity across cortical depths are sourced from Paquola et al., 2021^7^.

Each SANDI metric projected onto the cortical surface template displayed distinct microstructural patterns across 21 cortical depths, offering detailed insights into layer-specific microstructural organization. Figure 3 presents the intra-soma signal fraction *f*_*is*_ from the Connectome 2.0 scanner at different depths alongside the Merker staining intensity reflecting cell body density obtained from the BigBrain cytoarchitectonic atlas. Both profiles show the lowest values near the pial surface, followed by a gradual increase with increasing depth. Across all cortical regions, the intra-soma signal fraction *f*_*is*_ reached its peak at approximately 55% cortical depth, while the Merker staining intensity from the BigBrain cytoarchitectonic atlas peaked at around 63% depth.

**Figure 4:**
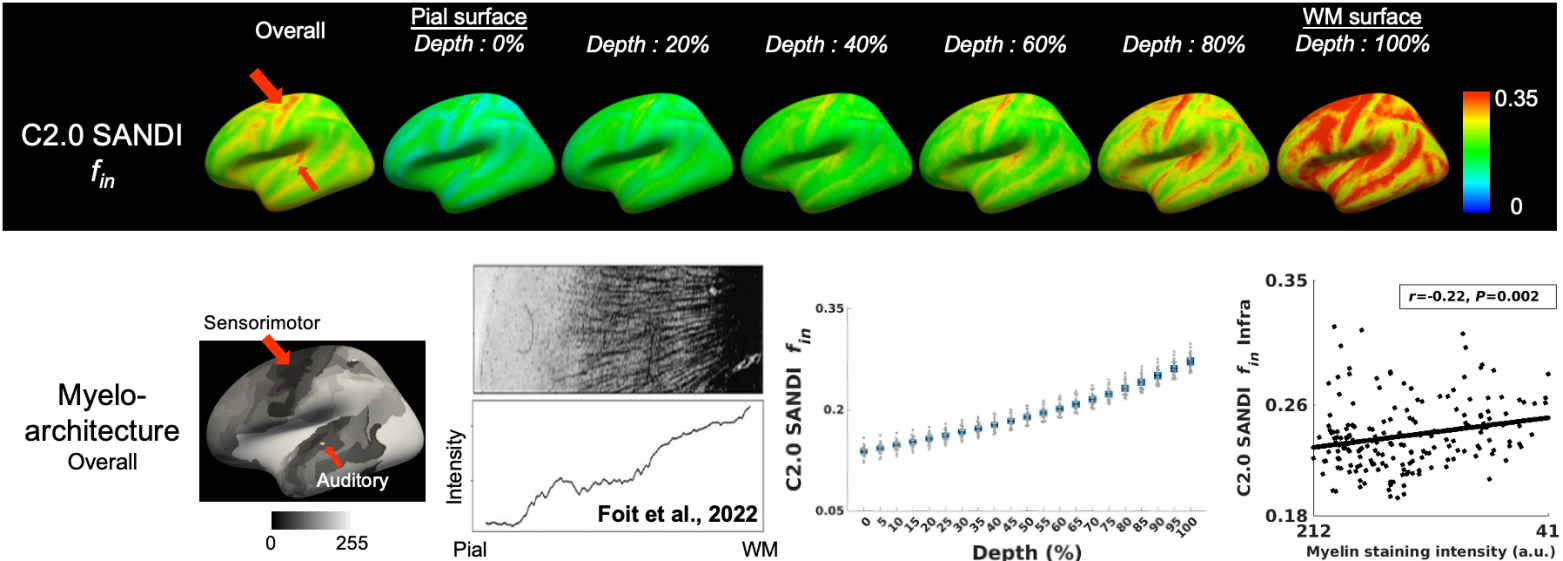
SANDI-derived intra-neurite signal fraction *f*_*in*_ on the Connectome 2.0 scanner, alongside the myelin staining data from the myeloarchitecture atlas across the cortical depths. In the myelin staining data, darker colors (i.e., lower intensity values) correspond to higher myelin concentration across regions defined by Nieuwenhuys’ parcellation. The data of myelin staining intensity across cortical depths and the figure of histology are sourced from Foit et al., 2022^8^, with permission from Elsevier.

The intra-neurite signal fraction *f*_*in*_ from the Connectome 2.0 scanner showed a progressive increase from the pial surface to the gray-white matter boundary, consistent with established histological observations (Figure 4). Among cortical areas, the sensorimotor and auditory cortices exhibited the highest intra-neurite signal fraction *f*_*in*_, in agreement with the myeloarchitectonic atlas (highlighted by red arrows). Furthermore, the intra-neurite signal fraction *f*_*in*_ in the infragranular layer was negatively correlated with myelin staining intensity across regions defined by Nieuwenhuys’ parcellation. (*r*=−0.22; *P*=0.002).

### Regional differences in intra-soma signal fraction *f*_*is*_ across cortical depths

**Figure 5:**
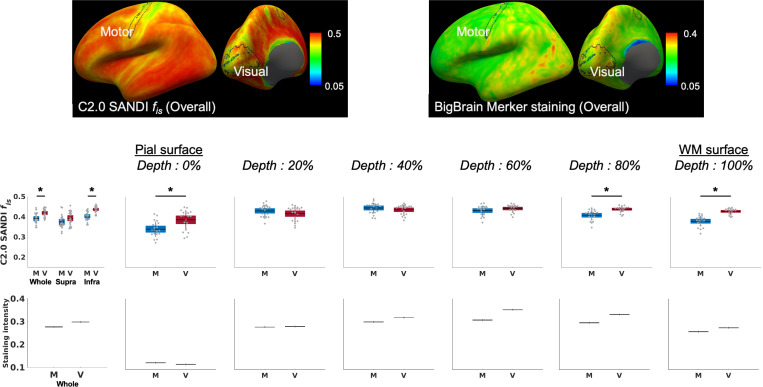
Comparison of SANDI-derived intra-soma signal fraction *f*_*is*_ between the motor cortex and visual cortex across the cortical depths, measured using the Connectome 2.0 scanner. Statistically significant differences are indicated (*: FDR-*P* < 0.05). The data of BigBrain Merker staining intensity across cortical depths are sourced from Paquola et al., 2021^7^.

When comparing the motor and visual cortices across multiple cortical depths (Figure 5), the intra-soma signal fraction *f*_*s*_ from the Connectome 2.0 scanner was higher in the visual cortex than in the motor cortex at specific depths, particularly in the deeper cortical depths, which aligns with Merker staining intensity from the BigBrain cytoarchitectonic atlas. Significant differences were observed in the full cortical depth (FDR-*P*<0.001) and within the infragranular layer (FDR-*P*<0.001), whereas no significant differences emerged in the supragranular layer. Specifically, the visual cortex exhibited significantly higher intra-soma signal fraction *f*_*is*_ at 0% (FDR-*P*=0.002), 80% (FDR-*P*<0.001), and 100% (FDR-*P*<0.001) cortical depths. In contrast, no significant differences were found between the two regions at 20%, 40%, and 60% depths.

### Relationship between intra-soma signal fraction *f*_*is*_ and cortical curvature

**Figure 6:**
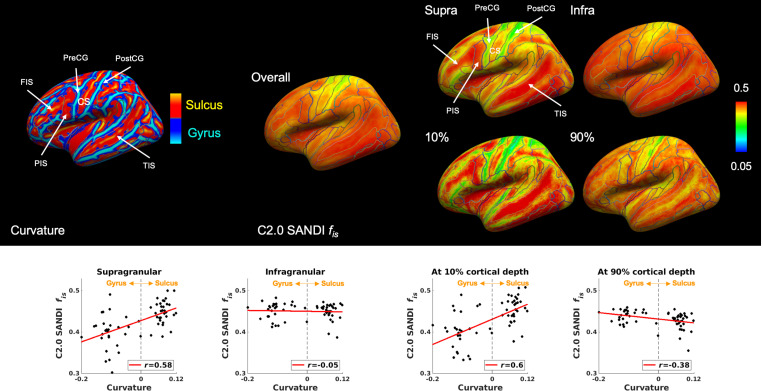
Relationship between intra-soma signal fraction *f*_*is*_ and cortical curvature. Cortical surface maps show curvature (left) and intra-soma signal fraction *f*_*is*_ (right) from the Connectome 2.0 scanner across overall, supragranular, and infragranular layers, as well as at 10% and 90% cortical depths, with labels from the aparc.a2009s.annot parcellation. Gyral regions are shown in blue (negative) and sulcal regions in red (positive) on the curvature map. CS: central sulcus; FIS: frontal inferior sulcus; PIS: parietal inferior sulcus; PostCG: postcentral gyrus; PreCG: precentral gyrus; TIS: temporal inferior sulcus.

The intra-soma signal fraction *f*_*is*_ exhibited a layer-specific association with cortical curvature revealing distinct distributions across gyral and sulcal regions, as shown in Figure 6. Correlation analyses confirmed this pattern, with a positive relationship observed in the supragranular layer (*r*=0.58; *P*<0.001), indicating higher intra-soma signal fraction *f*_*is*_ in sulcal fundi compared to gyral crowns. In contrast, a weak negative correlation was observed in the infragranular layer (*r*=−0.05; *P*=0.74), and a stronger negative correlation emerged at 90% cortical depth (*r*=−0.38; *P*=0.003), demonstrating higher intra-soma signal fraction *f*_*s*_ in the gyrus at deeper cortical layers.

## Discussion

We have advanced the *in vivo* characterization of human cortical microstructure by combining the high-performance gradient system of the next-generation Connectome 2.0 scanner with the SANDI model to resolve cytoarchitectonic and myeloarchitectonic features across cortical depths. We identified distinct laminar and regional microstructural profiles that closely aligned with established histological atlases^8,56,57^. The Connectome 2.0 scanner demonstrated improved sensitivity to restricted water diffusion within the neuritic compartment compared to the Connectome 1.0 scanner. We were able to detect layer-specific patterns of the SANDI-derived microstructural metrics, such as peak intra-soma signal fraction *f*_*is*_ at mid-cortical depth and increasing intra-neurite signal fraction *f*_*in*_ toward the gray-white matter boundary, along with regionally specific variations, including higher intra-soma signal fraction *f*_*is*_ in the visual cortex than in the motor cortex at deeper cortical depths. Moreover, a depth-dependent relationship between intra-soma signal fraction *f*_*is*_ and cortical curvature emerged, with higher intra-soma signal fraction *f*_*is*_ in sulci within the supragranular layer that shifted toward the gyri in deeper layers. Collectively, these findings suggest the promise of using high-performance gradient dMRI for detailed mapping of cortical microstructure *in vivo,* bridging the gap between traditional postmortem histology and *in vivo* neuroimaging.

In a direct comparison of SANDI metrics across the entire cortex, overall spatial patterns were largely consistent between the Connectome 2.0 scanner and its predecessor, Connectome 1.0. However, the Connectome 2.0 scanner demonstrated superior performance in detecting neuritic microstructure. By leveraging a higher *G*_*max*_, it enabled to achieve shorter diffusion and echo times during acquisition, which improved sensitivity to restricted water diffusion within neurites and resulted in significantly higher intra-neurite signal fraction *f*_*in*_. Shorter diffusion times strategically mitigate water exchange effects between the compartments^61–64^, which the SANDI model does not account for^51^. Although we hypothesized that the intra-soma signal fraction *f*_*is*_ would be higher with the Connectome 2.0 scanner, no significant difference was observed between the scanners. Nevertheless, these findings underscore the potential of the Connectome 2.0 scanner to provide a nuanced view of brain tissue microstructure, particularly myeloarchitecture, enabling a more sensitive assessment of subtle variations in cellular architecture associated with aging and neurodegenerative diseases.

Cortical depth-dependent dMRI analysis offers a more detailed examination of cortical regions^65–67^, unveiling microstructural information that may be overlooked by conventional ROI-based methods. SANDI metrics obtained with high-performance gradient systems revealed distinct profiles across cortical depths, providing a non-invasive characterization of the cortical laminar organization. The intra-soma signal fraction *f*_*is*_ and intra-neurite signal fraction *f*_*in*_ exhibited patterns that qualitatively aligned with known histological features of cortical layers. Specifically, our results showed a peak in intra-soma signal fraction *f*_*is*_ in the mid-cortical regions at approximately 55% cortical depth, corresponding to the peak in cell body density reported in the BigBrain atlas^7^, which occurred at 63% depth in layers enriched with large pyramidal neurons and higher cell body density. Intra-neurite signal fraction *f*_*in*_ progressively increased toward deeper cortical layers, consistent with histological data showing higher myelinated fiber density near the gray-white matter boundary^8^.

Region-specific variations in SANDI metrics further demonstrated the utility of a depth-dependent approach, as cytoarchitectonic and myeloarchitectonic properties are not uniform across the cortex. For instance, the primary motor cortex lacks a well-defined granular layer (layer IV), which deviates from the classical six-layered structure seen in somatosensory cortices^9^, while the primary visual cortex is marked by the line of Gennari, a dense band of myelination within layer IV^3,6^. Although precise *in vivo* delineation of such cytoarchitectonic and myeloarchitectonic distinctions remains challenging, our results showed a higher intra-soma signal fraction *f*_*is*_ in the visual cortex compared to the motor cortex, aligning with established neuroanatomical principles of cell density^68,69^. This difference was particularly pronounced at deeper cortical depths (80% and 100%), corresponding to the infragranular layers, where the Merker staining intensity from the BigBrain atlas showed a similar trend. In contrast, no significant differences in intra-soma signal fraction *f*_*is*_ were observed between the two regions at intermediate depths (20%, 40%, and 60%) or within the supragranular layers, further emphasizing the importance of depth-dependent analysis in capturing laminar-specific microstructural differences across cortical areas.

The highest intra-neurite signal fraction *f*_*in*_ values were detected in primary sensory areas, including somatosensory and auditory cortices, and in the primary motor cortex, aligning with neurite density estimates from another biophysical dMRI model, Neurite Orientation Dispersion and Density Imaging (NODDI), and with patterns reported in the myeloarchitectonic atlas^8,70^. These findings were further supported by a significant negative correlation between intra-neurite signal fraction *f*_*in*_ and myelin staining intensities across regions defined by Nieuwenhuys’ parcellation. However, the strength of this correlation was lower than anticipated, likely due to the definition of intra-neurite signal fraction *f*_*in*_ in the SANDI model, which captures water diffusion within both myelinated and unmyelinated axons, as well as dendrites. The inclusion of unmyelinated intra-neurite components reduces the specificity of the intra-neurite signal fraction *f*_*in*_ to myelinated fiber content, thereby attenuating the observed correlation with histological myelin density.

Our findings revealed a layer-specific relationship between intra-soma signal fraction *f*_*is*_ and cortical curvature, providing additional evidence for laminar variation in cytoarchitecture. Specifically, intra-soma signal fraction *f*_*is*_ was positively correlated with curvature in the supragranular layer, indicating higher intra-soma signal fraction *f*_*is*_ values in sulcal fundi compared to gyral crowns. In contrast, intra-soma signal fraction *f*_*is*_ showed a negative correlation with curvature in deeper cortical regions, where higher intra-soma signal fraction *f*_*is*_ values were observed in gyral regions. These results were further confirmed by a two-sample t-test between the sulcus and gyrus, with corresponding *P*< 0.001 at 10% depth and *P*= 0.008 at 90% depth. This inversion pattern is consistent with previous histological studies reporting neuronal density in supragranular and infragranular layers of gyri and sulci^71^. While our label-based analysis used the aparc.a2009s parcellation to define sulcal and gyral regions, curvature alone does not always align perfectly with anatomical definitions. For example, secondary folds at the base of deep sulci may exhibit locally positive curvature, typically associated with gyral crowns, despite being embedded within clearly sulcal cortex. Whether such regions more closely resemble sulcal or gyral cortex in their microstructural and cytoarchitectonic features remains an open question. This anatomical ambiguity should be taken into account when interpreting vertex-based analyses of curvature and its relationship to laminar architecture.

We further demonstrated the potential of cortical depth-dependent diffusion MRI to differentiate adjacent cortical regions by revealing distinct supragranular and infragranular intra-soma signal fraction *f*_*is*_ patterns that were not captured by conventional whole-thickness measures. Specifically, while overall intra-soma signal fraction *f*_*is*_ did not differ between BA4a and BA4p in the motor cortex, layer-specific comparisons uncovered a dissociation, with lower intra-soma signal fraction *f*_*is*_ in BA4a relative to BA4p in the supragranular layer, and higher intra-soma signal fraction *f*_*is*_ in BA4a in the infragranular layer. These opposing laminar profiles were obscured in regionally averaged metrics, highlighting the importance of laminar-specific analysis for resolving subtle cytoarchitectonic boundaries^72,73^. Taken together, the observed laminar patterns, region- and curvature-dependent variations and the ability to distinguish cytoarchitectonically adjacent areas underscore the power of layer- and region-specific SANDI metrics to capture microstructural and geometric features of the cortex. These findings highlight the promise of high-resolution, depth-dependent diffusion MRI as a noninvasive tool for detecting subtle, layer-specific cellular and neuritic alterations.

To bridge the gap between *in vivo* imaging and histological ground truth, we compared SANDI-derived microstructural metrics with established cytoarchitectonic and myeloarchitectonic atlases. This comparison provides a pathway toward validating dMRI-based measurements and interpreting the findings in the context of well-characterized cortical microstructural patterns. Our results demonstrated strong qualitative agreement between SANDI metrics and histological references, suggesting that SANDI can approximate the cortical microstructural features reflected in histology. However, it is important to note the limitations in establishing a direct, one-to-one correspondence between the two modalities, particularly given regional variations in complex geometry and inter-individual variability. In this study, two widely used histological atlases were employed to contextualize SANDI metrics: the BigBrain atlas for cytoarchitecture and the Nieuwenhuys atlas for myeloarchitecture. While informative, these references are limited, especially the Bigbrain atlas, which is based on a single 65-year-old brain and therefore lacks population-level generalizability^56^. This limitation complicates accurate alignment with *in vivo* data, potentially impacting the accuracy of cortical layer mapping and interpretation. Further investigation is required to improve the biological interpretability of cortical microstructure mapping using advanced dMRI techniques.

While the SANDI model provides valuable insights into cortical microstructure, it has inherent limitations in modeling microstructural properties. The model’s signal fractions are relative estimates derived from complex mathematical representations of water diffusion across three compartments, influenced by *T*_*2*_ weighting and linear interdependence among compartments, i.e., the sum of all signal fractions is 1^74^. A key challenge involves distinguishing between intra-soma and extracellular water compartments that share similar geometric models but exhibit different diffusivities. Additionally, the current model cannot fully account for the presence of free water or partial volume effects from adjacent cerebrospinal fluid, which can lead to biased estimates, particularly near cortical boundaries or pathologies with edema and inflammation^75,76^. Furthermore, partial volume effects arising from cortical folding, especially in highly curved regions, and limited spatial resolution can lead to mixing of signals across adjacent layers or tissue compartments, potentially biasing compartment-specific signal fraction estimates at the vertex level. To improve specificity, future research should focus on refining the model to better separate extracellular compartments (intermediate diffusivity, hindered diffusion) from soma (low diffusivity, restricted diffusion) and free water (high diffusivity, free diffusion), thereby reducing partial volume effects and enabling more accurate capturing of the cellular characteristics in the normal cortex and in pathological conditions.

Translating advances in gradient performance and biophysical modeling of dMRI signals into routine clinical practice remains an ongoing challenge. Although the ability to noninvasively characterize layer-specific cytoarchitecture and myeloarchitecture *in vivo* holds great promise for clinical applications, widespread implementation is limited by the high gradient strengths required, which are not yet standard in most clinical MRI systems. Encouragingly, recent studies have demonstrated the feasibility of applying the SANDI model on conventional 3T scanners, such as the Philips Ingenia CX and Siemens Magnetom Prisma, indicating that some meaningful microstructural information can still be extracted in patient populations despite hardware constraints^77,78^. Continued efforts are needed to optimize acquisition strategies, improve model robustness, and validate microstructural metrics across diverse populations and clinical conditions. Ultimately, these advancements could facilitate the routine clinical adoption of microstructural dMRI techniques for early diagnosis and longitudinal monitoring of neurodegenerative and psychiatric diseases. Furthermore, the integration of high-performance gradient systems into clinical imaging environments^27^ would significantly enhance the sensitivity, specificity, and spatial precision of these techniques, thereby expanding their diagnostic utility and impact.

## Conclusions

Our study shows the potential of *in vivo* brain tissue microstructural imaging achieved through combining ultra-high-performance gradients with the SANDI dMRI biophysical model. Using the next-generation Connectome 2.0 scanner, cortical depth-dependent analysis of SANDI metrics provides a detailed characterization of cortical cytoarchitecture and myeloarchitecture in the living human brain, revealing distinct profiles that align with established histological patterns, particularly in the cell body density distribution and myelinated fibers across cortical layers. Overall, our findings provide evidence that high-performance gradient systems can help bridge the gap between traditional postmortem histology and *in vivo* neuroimaging, paving the way for developing noninvasive biomarkers to detect subtle, layer-specific microstructural changes relevant to a wide variety of neurological and psychiatric disorders.

## Methods and Materials

### Participant Recruitment

We recruited young healthy adults between the ages of 19 and 40 years for scans on the Connectome 1.0 and Connectome 2.0 scanners. Participants for the Connectome 1.0 scans were recruited between September 2016 and June 2023. After the Connectome 1.0 scanner was then decommissioned and replaced by the Connectome 2.0 scanner in the same imaging scanner bay, a new group of participants was recruited for scans on the Connectome 2.0 scanner between September 2023 and October 2024. Our screening process excluded individuals with any history of neurological and psychiatric conditions, encompassing conditions such as dementia, cerebrovascular disease, brain tumors, head injuries, and any other central nervous system disorders. All subjects provided written informed consent prior to participation. The research protocols were reviewed and approved by the Institutional Review Board of Massachusetts General Brigham and were conducted in accordance with the Declaration of Helsinki.

### Data acquisition

All MRI scans were performed at the Athinoula A. Martinos Center for Biomedical Imaging, Massachusetts General Hospital. The 3T Connectome 2.0 MRI scanner (MAGNETOM Connectom.X, Siemens Healthineers, Erlangen, Germany) is equipped with a *G*_max_ of 500 mT/m and an *SR*_max_ of 600 T/m/s, using a custom-built 72-channel *in vivo* head coil for signal reception^79^. The 3T Connectome 1.0 MRI scanner (MAGNETOM Connectom, Siemens Healthcare) was equipped with a *G*_max_ of 300 mT/m and *SR*_max_ of 200 T/m/s using a custom-built 64-channel *in vivo* head coil^80^. On both scanners, dMRI data were acquired using a pulsed gradient spin-echo echo-planar-imaging (EPI) sequence. The diffusion times were set to the minimum accessible values for each system for the maximum b-value of 6000 s/mm^2^, with D = 13 ms on the Connectome 2.0 scanner and 19 ms on the Connectome 1.0 scanner, respectively, and diffusion-weighted gradient durations (*δ*) of 6 ms and 8 ms, respectively. A total of eight *d*-values were linearly sampled in gradient strength up to *G*_max_, with 32 diffusion encoding directions for b < 2400 s/mm^2^ (b = 50, 350, 800, and 1500 s/mm^2^) and 64 directions for b ≥ 2400 s/mm^2^ (b = 2400, 3450, 4750, and 6000 s/mm^2^) uniformly distributed on a sphere. Interspersed non-diffusion-weighted images (b = 0 s/mm^2^) were obtained for every 16 diffusion-weighted images to normalize signal intensity. The repetition time/echo time (TR/TE) were 3600/53 ms for the Connectome 2.0 scanner and 4000/77 ms for the Connectome 1.0 scanner. The imaging planes for the Connectome 2.0 scanner were axial, whereas those for the Connectome 1.0 scanner were sagittal. Additional common parameters for both scanners included: 2 mm isotropic voxel size, partial Fourier = 6/8, generalized autocalibrating partially parallel acquisition (GRAPPA) acceleration factor = 2, simultaneous multislice (SMS) acceleration factor = 2, anterior-to-posterior phase encoding direction, and adaptive coil combination. To correct for susceptibility-induced distortion, we acquired ten additional non-diffusion-weighted images at the beginning of the dMRI scans with a reversed-phase encoding direction (posterior-to-anterior).

For cortical surface reconstruction and segmentation, high-resolution 3D *T*_1_-weighted anatomical images were acquired during the same session. For the Connectome 2.0 scanner, a magnetization-prepared rapid acquisition with gradient echo (MPRAGE) sequence was employed with the imaging parameters: 1 mm isotropic voxel size, TR/ TE = 2500/3.36 ms, TI = 1100 ms, flip angle = 8 °, and GRAPPA acceleration factor = 2. For the Connectome 1.0 scanner, we used a multi-echo magnetization-prepared rapid acquisition with gradient echo (MEMPRAGE) sequence with the following parameters: 1 mm isotropic voxel size, TR/TE = 2530/1.15, 3.03, 4.89, and 6.75 ms, TI = 1100 ms, flip angle = 7 ^o^, and GRAPPA acceleration factor = 3.

### Data processing

dMRI data were preprocessed using an in-house script based on the DESIGNER pipeline^81^. Raw dMRI data were corrected for Gibbs ringing artifact using the “mrdegibbs” function in MRtrix3^82,83^, susceptibility and eddy current-induced distortions using the “topup” and “eddy” functions in FSL (https://fsl.fmrib.ox.ac.uk)^84,85^, followed by a gradient non-linearity correction^86^. A self-similarity-based super-resolution image processing technique was applied to dMRI data by introducing high-resolution details from *T*_*1*_-weighted anatomical images^58–60^, resulting in 1 mm high-resolution dMRI data to reduce partial volume effects (See Supplementary Note 1 for details). The SANDI model was fitted to multi-shell dMRI signals averaged over gradient directions (spherical mean), employing the SANDI MATLAB toolbox (https://github.com/palombom/SANDI-Matlab-Toolbox-v1.0)^51^, with intrinsic soma diffusivity *(Dis)* fixed at 2 μm^2^/ms^87^. To assess the robustness of parameter estimation, a noise propagation analysis was conducted using simulated signals incorporating Rician noise as shown in the Supplementary Note 2. Resulting SANDI parametric maps comprise the intra-soma signal fraction *(f*_*is*_*),* intra-neurite signal fraction *(f*_*in*_*),* extracellular signal fraction (*f*_*ec*_), apparent soma radius (*R*_*s*_), intra-neurite diffusivity *(D*_*in*_*),* and extracellular diffusivity (*D*_*ec*_). In this study, the intra-soma signal fraction *f*_*is*_ and intra-neurite signal fraction *f*_*in*_ *were* of particular interest, as they reflect distinct aspects of the underlying tissue microstructure. Specifically, intra-soma signal fraction *f*_*is*_ reflects cytoarchitectonic features such as cell body density and organization, while intra-neurite signal fraction *f*_*in*_ is associated with the myeloarchitecture, particularly the distribution of aligned neurites, including axons and dendrites.

We processed 3D *T*_*1*_-weighted anatomical images using FreeSurfer (version 7.1.4, https://surfer.nmr.mgh.harvard.edu) through the standard “recon-all” pipeline for skull stripping, cortical gray matter parcellation, and cortical surface reconstruction^88^. From this pipeline, we obtained cortical curvature maps and the aparc.a2009s.annot parcellation, as well as additional surface-based labels for regions of interest. The primary motor was defined based on Brodmann’s area 4, comprising its anterior (4a) and posterior (4p) subdivisions. The visual cortex was delineated using Brodmann area 17 (primary visual cortex, V1) and area 18 (secondary visual cortex, V2).

The averaged non-diffusion-weighted image was aligned with the *T*_*1*_-weighted anatomical image using the “bbregister” function in FreeSurfer, which employs a boundary-based rigid body transformation with 6 degrees of freedom. To investigate laminar patterns of cortical organization, the cortex was segmented into supragranular layer and infragranular layers using a deep learning-based approach guided by cortical curvature and gray matter structure^89^, with surface placement based on an optimized isovolume model. A cascaded multi-resolution U-Net was trained on *in vivo* and *ex vivo* MRI channels, with weak supervision applied to the *in vivo* data to ensure that the combined supragranular and infragranular layers fully covered the cortical gray matter labels. We then utilized the FreeSurfer commands “mri_compute_layer_fractions” and “mri_compute_layer_intensities” to extract SANDI-derived metrics across the whole cortical thickness as well as within supragranular and infragranular layers. The cortex was further divided into 21 evenly spaced depth intervals (5% intervals), ranging from the pial surface (0% depth) to the white matter boundary (100% depth), for a depth-dependent analysis of microstructural features. SANDI metrics were extracted at these depths using the “mri_vol2surf” function in FreeSurfer based on the transformation information from the co-registration between the non-diffusion-weighted image and the *T*_*1*_-weighted image. Individual subject data were aligned to the FreeSurfer “fsaverage” cortical surface template using the FreeSurfer “mri_vol2surf” function. For visualization, we averaged the SANDI metrics on the template space across individuals scanned on each scanner.

### Histological atlas

Histological atlases, which display comprehensive detail on cortical cytoarchitecture and myeloarchitecture, served as references for our *in vivo* SANDI metrics. For cytoarchitecture, the BigBrain atlas was employed, which features a high-resolution 3D reconstruction of a human brain created from histological sections stained for cell bodies using the Merker technique^56,57^. The dataset provides a microscopic perspective on cell body density and its laminar patterns throughout the entire cortex. Myeloarchitectonic information was derived from the atlas developed by Nieuwenhuys et al^90^. Based on the high-resolution mapping of cortical myelination patterns, this atlas presents the distribution and density of myelinated nerve fibers across different cortical areas and layers. To enable spatial correspondence with SANDI-derived microstructural metrics, we used cortical surface-based versions of the BigBrain cytoarchitectonic atlas (for intra-soma signal fraction, *f*_*is*_*)* and the myeloarchitecture atlas (for intra-neurite signal fraction, *f*_*in*_*)*, both aligned to the FreeSurfer “fsaverage” template space^7,8^.

### Statistical analysis

All statistical analyses were performed using MATLAB (version 9.13, MathWorks, Natick, MA, USA). For the statistical analysis, the SANDI-derived microstructural metrics *(f*_*is*_ and *f*_*in*_*)* were extracted from each subject’s native space. The Kolmogorov-Smirnov test was used to assess data normality. To compare SANDI metrics across scanners, two-sample t-tests were performed on values averaged across the full cortical depth of the entire cortex between the Connectome 2.0 and Connectome 1.0 scanners. To take advantage of the ultra-high-gradient strength of the Connectome 2.0 scanner, all subsequent analyses comparing SANDI metrics to histological atlases and prior cytoarchitectonic and myeloarchitectonic studies were performed exclusively using data from the Connectome 2.0 scanner. Paired t-tests were used to compare intra-soma signal fractions *f*_*is*_ and intra-neurite signal fractions *f*_*in*_ between the supragranular and infragranular layers. Pearson’s correlation analysis was used to assess the correlation between intra-neurite signal fractions *f*_*in*_ in the infragranular layer and myelin staining intensity from the myeloarchitecture atlas^8^, using cortical labels defined by Nieuwenhuys’ parcellation^90^, excluding labels with missing or very low-intensity data. The infragranular layer was specifically examined because it contains a relatively high density of myelinated axons, which contribute significantly to the intra-neurite signal *f*_*in*_. Additional paired t-tests assessed regional differences in the intra-soma signal fractions *f*_*is*_ between the motor and visual cortices. For each subject, the median intra-soma signal fractions *f*_*is*_ value within each region was used due to the relatively small size of the regions of interest. Comparisons were performed under the following conditions: 1) across the entire cortical depths, 2) within the supragranular layer, 3) within the infragranular layer, and 4) at specific cortical depths (0%, 20%, 40%, 60%, 80%, and 100%). To examine the layer-specific relationship between cortical curvature and intra-soma signal fractions *f*_*is*_, Pearson’s correlation analyses were performed separately in the supragranular and infragranular layers, as well as at 10% and 90% cortical depths. The significance threshold was set at *P*-value < 0.05, corrected for multiple comparisons using false discovery rate (FDR) correction.

## Supplementary Material

This is a list of supplementary files associated with this preprint. Click to download.

- Editorialpolicychecklist.pdf
- Reportingsummarydocument.pdf
- Supportinginformation.docx

## Data Availability

The data and code used in the study are available upon direct request as well as the conditions for its sharing or re-use.

## Abbreviations

dMRI: diffusion MRI
EPI: echo-planar-imaging
FDR: false discovery rate
*G*_max_: maximum gradient strength
GRAPPA: generalized autocalibrating partially parallel acquisition
MEMPRAGE: multi-echo magnetization-prepared rapid acquisition with gradient echo
MPRAGE: magnetization-prepared rapid acquisition with gradient echo
MRI: magnetic resonance imaging
NODDI: neurite orientation dispersion and density imaging
SANDI: soma and neurite density imaging
SMS: simultaneous multislice
*SR*_max_: maximum slew rate
TE: echo time
TR: repetition time
